# Supplementary material for: Resolving the full spectrum of human genome variation using Linked-Reads
Source: Genome Res. 2019 Apr;29(4):635–45. doi: 10.1101/gr.234443.118 (PMC6442396; doi:10.1101/gr.234443.118)
Supplement: Supplemental Material [file supp_gr.234443.118_Supplemental_longranger-2.2.zip › longranger-2.2.tar.bz2!/lr2.2-public/lib/stan/cmdstan/stan/doc/api/html/structstan_1_1math_1_1coupled__ode__system_3_01_f_00_01stan_1_1agrad_1_1var_00_01double_01_4.html]

Stan: stan::math::coupled\_ode\_system< F, stan::agrad::var, double > Struct Template Reference


|  |  |
| --- | --- |
|  | Stan  2.5.0  probability, sampling & optimization |


- Main Page
- Related Pages
- Namespaces
- Classes
- Files

- Class List
- Class Index
- Class Hierarchy
- Class Members

All Classes Namespaces Files Functions Variables Typedefs Enumerations Enumerator Friends Macros Pages

- stan
- math
- coupled\_ode\_system< F, stan::agrad::var, double >

Public Member Functions |
Public Attributes |
List of all members

stan::math::coupled\_ode\_system< F, stan::agrad::var, double > Struct Template Reference

The coupled ODE system for unknown initial values and known parameters.
More...

`#include <coupled_ode_system.hpp>`

|  |  |
| --- | --- |
| Public Member Functions | |
|  | coupled\_ode\_system (const F &f, const std::vector< stan::agrad::var > &y0, const std::vector< double > &theta, const std::vector< double > &x, const std::vector< int > &x\_int, std::ostream \*msgs) |
|  | Construct a coupled ODE system for an unknown initial state and known parameters givne the specified base system functor, base initial state, parameters, data, and an output stream for messages. More... |
|  | |
| void | operator() (const std::vector< double > &y, std::vector< double > &dy\_dt, double t) |
|  | Calculates the derivative of the coupled ode system with respect to the state y at time t. More... |
|  | |
| int | size () const |
|  | Returns the size of the coupled system. More... |
|  | |
| std::vector< double > | initial\_state () |
|  | Returns the initial state of the coupled system. More... |
|  | |
| std::vector< std::vector  < stan::agrad::var > > | decouple\_states (const std::vector< std::vector< double > > &y) |
|  | Return the solutions to the basic ODE system, including appropriate autodiff partial derivatives, given the specified coupled system solution. More... |
|  | |

|  |  |
| --- | --- |
| Public Attributes | |
| const F & | f\_ |
|  | |
| const std::vector  < stan::agrad::var > & | y0\_ |
|  | |
| std::vector< double > | y0\_dbl\_ |
|  | |
| const std::vector< double > & | theta\_dbl\_ |
|  | |
| const std::vector< double > & | x\_ |
|  | |
| const std::vector< int > & | x\_int\_ |
|  | |
| std::ostream \* | msgs\_ |
|  | |
| const int | N\_ |
|  | |
| const int | M\_ |
|  | |
| const int | size\_ |
|  | |

## Detailed Description

### template<typename F> struct stan::math::coupled\_ode\_system< F, stan::agrad::var, double >

The coupled ODE system for unknown initial values and known parameters.

If the original ODE has states of size N, the coupled system has N + N \* N states. (derivatives of each state with respect to each initial value)

The coupled system has N + N \* N states, where N is the size of the state vector in the base system.

The first N states correspond to the base system's N states:

The next N states correspond to the sensitivities of the initial conditions with respect to the to the first base system equation:

The next N states correspond to the sensitivities with respect to the second base system equation, etc.

Template Parameters
:   |  |  |
    | --- | --- |
    | F | type of base ODE system functor |

Definition at line 281 of file coupled\_ode\_system.hpp.

## Constructor & Destructor Documentation

template<typename F >

|  |  |  |  |
| --- | --- | --- | --- |
| stan::math::coupled\_ode\_system< F, stan::agrad::var, double >::coupled\_ode\_system | ( | const F & | *f*, |
|  |  | const std::vector< stan::agrad::var > & | *y0*, |
|  |  | const std::vector< double > & | *theta*, |
|  |  | const std::vector< double > & | *x*, |
|  |  | const std::vector< int > & | *x\_int*, |
|  |  | std::ostream \* | *msgs* |
|  | ) |  |  |

inline

Construct a coupled ODE system for an unknown initial state and known parameters givne the specified base system functor, base initial state, parameters, data, and an output stream for messages.

Parameters
:   |  |  |  |
    | --- | --- | --- |
    | [in] | f | base ODE system functor. |
    | [in] | y0 | initial state of the base ODE. |
    | [in] | theta | system parameters. |
    | [in] | x | real data. |
    | [in] | x\_int | integer data. |
    | [in,out] | msgs | output stream for messages. |

Definition at line 307 of file coupled\_ode\_system.hpp.

## Member Function Documentation

template<typename F >

|  |  |  |  |  |  |
| --- | --- | --- | --- | --- | --- |
| std::vector<std::vector<stan::agrad::var> > stan::math::coupled\_ode\_system< F, stan::agrad::var, double >::decouple\_states | ( | const std::vector< std::vector< double > > & | *y* | ) |  |

inline

Return the solutions to the basic ODE system, including appropriate autodiff partial derivatives, given the specified coupled system solution.

Parameters
:   |  |  |
    | --- | --- |
    | y | the vector of the coupled states after solving the ode |

Definition at line 430 of file coupled\_ode\_system.hpp.

template<typename F >

|  |  |  |  |  |
| --- | --- | --- | --- | --- |
| std::vector<double> stan::math::coupled\_ode\_system< F, stan::agrad::var, double >::initial\_state | ( |  | ) |  |

inline

Returns the initial state of the coupled system.

Because the starting state is unknown, the coupled system incorporates the initial conditions as parameters. The initial conditions for the coupled part of the system are set to zero along with the rest of the initial state, because the value of the initial state has been moved into the parameters.

Returns
:   the initial condition of the coupled system. This is a vector of length size() where all elements are 0.

Definition at line 418 of file coupled\_ode\_system.hpp.

template<typename F >

|  |  |  |  |
| --- | --- | --- | --- |
| void stan::math::coupled\_ode\_system< F, stan::agrad::var, double >::operator() | ( | const std::vector< double > & | *y*, |
|  |  | std::vector< double > & | *dy\_dt*, |
|  |  | double | *t* |
|  | ) |  |  |

inline

Calculates the derivative of the coupled ode system with respect to the state y at time t.

Parameters
:   |  |  |  |
    | --- | --- | --- |
    | [in] | y | the current state of the coupled ode system. This is a a vector of double of length size(). |
    | [out] | dy\_dt | a vector of length size() with the derivatives of the coupled system evaluated with state y and time t. |
    | [in] | t | time. |

Exceptions
:   |  |  |
    | --- | --- |
    | exception | if the system functor does not return a derivative vector of the same size as the state vector. |

Definition at line 341 of file coupled\_ode\_system.hpp.

template<typename F >

|  |  |  |  |  |
| --- | --- | --- | --- | --- |
| int stan::math::coupled\_ode\_system< F, stan::agrad::var, double >::size | ( |  | ) | const |

inline

Returns the size of the coupled system.

Returns
:   size of the coupled system.

Definition at line 400 of file coupled\_ode\_system.hpp.

## Member Data Documentation

template<typename F >

const F& stan::math::coupled\_ode\_system< F, stan::agrad::var, double >::f\_

Definition at line 283 of file coupled\_ode\_system.hpp.

template<typename F >

const int stan::math::coupled\_ode\_system< F, stan::agrad::var, double >::M\_

Definition at line 291 of file coupled\_ode\_system.hpp.

template<typename F >

std::ostream\* stan::math::coupled\_ode\_system< F, stan::agrad::var, double >::msgs\_

Definition at line 289 of file coupled\_ode\_system.hpp.

template<typename F >

const int stan::math::coupled\_ode\_system< F, stan::agrad::var, double >::N\_

Definition at line 290 of file coupled\_ode\_system.hpp.

template<typename F >

const int stan::math::coupled\_ode\_system< F, stan::agrad::var, double >::size\_

Definition at line 292 of file coupled\_ode\_system.hpp.

template<typename F >

const std::vector<double>& stan::math::coupled\_ode\_system< F, stan::agrad::var, double >::theta\_dbl\_

Definition at line 286 of file coupled\_ode\_system.hpp.

template<typename F >

const std::vector<double>& stan::math::coupled\_ode\_system< F, stan::agrad::var, double >::x\_

Definition at line 287 of file coupled\_ode\_system.hpp.

template<typename F >

const std::vector<int>& stan::math::coupled\_ode\_system< F, stan::agrad::var, double >::x\_int\_

Definition at line 288 of file coupled\_ode\_system.hpp.

template<typename F >

const std::vector<stan::agrad::var>& stan::math::coupled\_ode\_system< F, stan::agrad::var, double >::y0\_

Definition at line 284 of file coupled\_ode\_system.hpp.

template<typename F >

std::vector<double> stan::math::coupled\_ode\_system< F, stan::agrad::var, double >::y0\_dbl\_

Definition at line 285 of file coupled\_ode\_system.hpp.

---

The documentation for this struct was generated from the following file:

- src/stan/agrad/rev/ode/coupled\_ode\_system.hpp


---

[ Stan Home Page ]

*© 2011–2014,
Stan Development Team.*
